# Differential regulation of osteoclastogenesis by Notch2/Delta-like 1 and Notch1/Jagged1 axes

**DOI:** 10.1186/ar3758

**Published:** 2012-03-05

**Authors:** Chiyoko Sekine, Akemi Koyanagi, Noriko Koyama, Katsuto Hozumi, Shigeru Chiba, Hideo Yagita

**Affiliations:** 1 Department of Immunology, Juntendo University School of Medicine, 2-1-1 Hongo, Bunkyo-ku, Tokyo, 113-8421, Japan; 2 Division of Cell Biology, Biomedical Research Center, Juntendo University School of Medicine, 2-1-1 Hongo, Bunkyo-ku, Tokyo, 113-8421, Japan; 3 Probiotics Research Laboratory, Juntendo University School of Medicine, 2-1-1 Hongo, Bunkyo-ku, Tokyo, 113-8421, Japan; 4 Department of Immunology, Tokai University School of Medicine, 143 Shimokasuya, Isehara, Kanagawa, 259-1193, Japan; 5 Department of Clinical and Experimental Hematology, Graduate School of Comprehensive Human Sciences, University of Tsukuba, 1-1-1 Tennodai, Tsukuba, Ibaragi, 305-8575, Japan

## Abstract

**Introduction:**

Osteoclastogenesis plays an important role in the bone erosion of rheumatoid arthritis (RA). Recently, Notch receptors have been implicated in the development of osteoclasts. However, the responsible Notch ligands have not been identified yet. This study was undertaken to determine the role of individual Notch receptors and ligands in osteoclastogenesis.

**Methods:**

Mouse bone marrow-derived macrophages or human peripheral blood monocytes were used as osteoclast precursors and cultured with receptor activator of nuclear factor-kappaB ligand (RANKL) and macrophage-colony stimulating factor (M-CSF) to induce osteoclasts. Osteoclasts were detected by tartrate-resistant acid phosphatase (TRAP) staining. K/BxN serum-induced arthritic mice and ovariectomized mice were treated with anti-mouse Delta-like 1 (Dll1) blocking monoclonal antibody (mAb).

**Results:**

Blockade of a Notch ligand Dll1 with mAb inhibited osteoclastogenesis and, conversely, immobilized Dll1-Fc fusion protein enhanced it in both mice and humans. In contrast, blockade of a Notch ligand Jagged1 enhanced osteoclastogenesis and immobilized Jagged1-Fc suppressed it. Enhancement of osteoclastogenesis by agonistic anti-Notch2 mAb suggested that Dll1 promoted osteoclastogenesis via Notch2, while suppression by agonistic anti-Notch1 mAb suggested that Jagged1 suppressed osteoclastogenesis via Notch1. Inhibition of Notch signaling by a gamma-secretase inhibitor suppressed osteoclastogenesis, implying that Notch2/Dll1-mediated enhancement was dominant. Actually, blockade of Dll1 ameliorated arthritis induced by K/BxN serum transfer, reduced the number of osteoclasts in the affected joints and suppressed ovariectomy-induced bone loss.

**Conclusions:**

The differential regulation of osteoclastogenesis by Notch2/Dll1 and Notch1/Jagged1 axes may be a novel target for amelioration of bone erosion in RA patients.

## Introduction

Notch signaling pathways play key roles in cell-fate decision and differentiation in many tissues during embryonic and postnatal development [1]. Four mammalian Notch receptors have been identified, designated as Notch1 to Notch4. Interaction of Notch receptors with membrane-bound ligands of the Delta and Jagged families (Delta-like1 (Dll1), Dll4, Jagged1, and Jagged2) induces gamma-secretase-mediated cleavage and translocation of Notch intracellular domain (ICD) into the nucleus, where it interacts with the transcription factor CSL. Once bound to CSL, Notch intracellular domain recruits other coactivators, including mastermind proteins, and this transcriptional activation complex induces the expression of downstream target genes, such as *Hairly Enhancer of Split -1 (Hes-1) *[2].

The importance of Notch signaling in osteoclastogenesis has recently been reported [3,4]. Osteoclasts are derived from the monocyte/macrophage lineage and are responsible for bone resorption [5]. Osteoclast differentiation is a multistep process that leads to expression of tartrate-resistant acid phosphatase (TRAP), multinucleation and bone-resorbing activity. It has been demonstrated that receptor activator of nuclear factor-kappaB ligand (RANKL) and macrophage-colony stimulating factor (M-CSF) are critical for osteoclast development [6]. CD51/CD61, TRAP and matrix metalloproteinase-9 are widely used as specific markers for osteoclasts [7]. Controlling osteoclastogenesis is important for bone homeostasis and an abnormal osteoclastogenesis leads to imbalance of bone remodeling that is related to various diseases such as osteoporosis, rheumatoid arthritis (RA), and multiple myeloma [5].

RA is a chronic autoimmune disease characterized by inflammation of synovial joints leading to erosion of bone and ultimately functional loss of joints. This bone destruction is caused by enhanced activity of osteoclasts [8]. In chronic inflammation, pro-osteoclastogenic factors often predominate, leading to increased osteoclast formation and pathological bone resorption. Current therapies to treat RA have focused on inhibition of inflammation in the joints. To prevent structural destruction of the joints, it is important to explore the regulation of osteoclasts as a new therapeutic approach for the treatment of RA.

A recent report demonstrated that deletion of *Notch1 *and/or *Notch3 *in mouse osteoclast precursor cells promoted osteoclast differentiation and overexpression of a Notch ligand Jagged1 suppressed osteoclastogenesis, suggesting a suppressive role for Notch/Jagged1 in osteoclastogenesis [3]. On the other hand, Notch2 has been shown to accelerate osteoclastogenesis in association with nuclear factor-kappaB, and induction of Notch signaling by Jagged1 promoted osteoclast differentiation [4]. Thus, possibly differential contributions of individual Notch receptors and ligands to the regulation of osteoclastogenesis remain elusive. In addition, the contribution of Notch receptors and ligands to human osteoclastogenesis has not been determined yet.

We have recently established a panel of monoclonal antibodies (mAbs) specific for mouse Notch receptors and ligands [9]. In this study, we investigated the effect of these mAbs on the differentiation of bone marrow (BM) cells into osteoclasts. We have also newly established mAbs against human Notch receptors and ligands, and determined their effects on the osteoclastogenesis from human peripheral blood monocytes (PBmono). Our results suggest that Dll1/Notch2 interaction promotes osteoclastogenesis, whereas Jagged1/Notch1 interaction suppresses it in both mice and humans. Actually, treatment with anti-mouse Dll1 blocking mAb ameliorated K/BxN serum-induced arthritis, a mouse model of RA, and reduced osteoclasts number in the affected joints. The differential regulation of osteoclastogenesis by the Dll1/Notcn2 and Jagged1/Notch1 axes may have pathological and therapeutic relevancies to RA.

## Materials and methods

### Mice

C57BL/6 mice were purchased from Charles River (Oriental Yeast, Tokyo, Japan). Dll1 conditional knockout mice were generated as described previously [10]. For inducible deletion of Dll1, four-week-old Dll1^lox/lox ^Mx-Cre^+ ^or littermate control Dll1^lox/lox ^Mx-Cre^- ^mice were injected with 0.3 mg of poly(I):(C) twice a week for two weeks, and used three weeks later. All animal experiments were approved by Juntendo University Animal Experimental Ethics Committee.

### Reagents

The gamma-secretase inhibitor DAPT (N-[N-(3,5-difluorophenacetyl-L- alanyl)]-S-phenylglysin t-butyl ester), was purchased from Calbiochem (San Diego, CA, USA). The mouse Jagged1-Fc and mouse Dll1-Fc fusion proteins were generated as previously described [11]. The mouse Jagged2-Fc and human Jagged1-Fc fusion proteins were purchased from R&D Systems (Minneapolis, MN, USA). The human Dll1-Fc fusion protein was purchased from Alexis Biochemicals (Lausen, Switzerland). The human IgG Fc fragment was purchased from Acris Antibodies (Herford, Germany). Mouse Fc Block (2.4G2; BD Bioscience, San Jose, CA, USA) and Functional Grade Purified Human Fc(gamma)R-Binding Inhibitor (eBioscience, San Diego, CA, USA) were used to block non-specific binding of mAbs to Fc(gamma) receptors.

### Generation of mAbs

To generate the mAbs specific for human Dll1, Dll4, Jagged1 and Jagged2, Balb/c mice (Charles River) were immunized by intraperitoneal injection of human Dll1-Fc or Jagged1-Fc fusion protein, recombinant human Dll4 (R&D Systems), or Jagged2-transfected CHO cells three times at seven-day intervals. Three days after the final immunization, the splenocytes were fused with P3U1 myeloma cells. After hypoxanthine aminopterin thymidine (HAT) selection, antibodies that react with human Dll1-, Dll4-, Jagged1- or Jagged2-transfected CHO cells, but not with untransfected CHO cells, were screened by flow cytometry. Each mAb was cloned by limiting dilution.

Balb/c mice were immunized by intraperitoneal injection of human Notch1-Fc, Notch2-Fc or Notch3-Fc fusion protein (R&D Systems), or Notch4-transfected CHO cells three times at seven-day intervals. The hybridoma cells were prepared as described above, and antibodies that react with human Notch1-, Notch2-, Notch3- or Notch4-transfected CHO cells, but not with untransfected CHO cells, were screened and cloned. All these mAbs were purified from ascites produced in pristan-primed ICR nude mice by the caprylic acid and ammonium sulfate precipitation method [12] and labeled with biotin for flow cytometric analysis.

### Other antibodies

Generation and characterization of hamster IgG mAbs specific for mouse Notch1 (HMN1-12), Notch2 (HMN2-29), Notch3 (HMN3-133), Notch4 (HMN4-14), Dll1 (HMD1-5), Dll4 (HMD4-2), Jagged1 (HMJ1-29) and Jagged2 (HMJ2-1) have been described in our recent papers [9,13]. Stimulating activity of the anti-receptor mAbs and blocking activity of the anti-ligand mAbs have been verified *in vitro *or *in vivo *[9,13-20]. Fluorescein isothiocyanate (FITC) -labeled mAbs against mouse CD11c (HL3), mouse CD61 (2C9.G3), human CD11c (3.9) and human CD51/CD61 (23C6), PE-labeled mAb against mouse CD11b (M1/70), and human CD14 (61D3), APC- or PE-conjugated streptavidin were obtained from eBioscience. FITC-labeled mAb against mouse F4/80 (CI:A3-1) was from CALTAG (Carlsbad, CA, USA). Abs specific for cleaved-Notch1 (Val1744) and Notch2 intracellular domain (411801) were purchased from Cell Signaling (Beverly, MA, USA) and R&D Systems, respectively. Ab against TATA binding protein (TBP, ab63766) was from Abcam (Cambridge, MA, USA).

### Cell culture

Murine femoral BM cells from C57BL/6 mice were cultured with 50 ng/ml recombinant mouse (rm) M-CSF (Wako Chemicals, Osaka, Japan) in alpha-MEM media (GIBCO, Grand Island, NY, USA) containing 10% fetal bovine serum (FBS; JRH Bioscience, Lenexa, KS, USA) for 72 hours. Then, adherent cells were used as osteoclast precursors (> 90% CD11b^+^; Day 0). These cells (1 × 10^5 ^cells/ml) were cultured in the presence of 20 ng/ml rmM-CSF and 100 ng/ml recombinant human (rh) RANKL (Wako Chemicals). On Day 5, cultures were fixed with 3.7% formaldehyde and osteoclasts were characterized by staining for TRAP activity (TRAP staining kit, Primary Cell Co., Hokkaido, Japan). TRAP-positive multinucleated cells (MNCs) were observed under a microscope and counted as osteoclasts. The numbers of osteoclasts were analyzed statistically by unpaired Student's *t *test.

Peripheral blood mononuclear cells were prepared from heparinized blood, obtained from healthy individuals with informed consent, by Ficoll-Hypaque density gradient centrifugation. Peripheral blood mononuclear cells were suspended in alpha-MEM media supplemented with 10% FBS and cultured for one hour in a flask. Nonadherent cells were then removed by washing the flask twice with phosphate-buffered saline (PBS). Adherent cells (> 92% CD14^+^) were used as osteoclast precursors (PBmono). These cells were further cultured in alpha-MEM media supplemented with 10% FBS in the presence of 50 ng/ml rhM-CSF (HumanZyme, Chicago, IL, USA) for 72 hours (Day 0). Then, cells (5 × 10^5 ^cells/ml) were cultured in the presence of 20 ng/ml rhM-CSF and 100 ng/ml rhRANKL (Wako Chemicals). On Day 5, osteoclasts were characterized by staining for TRAP activity. The numbers of osteoclasts were counted and analyzed statistically by unpaired Student's *t *test.

### Flow cytometry

Multi-color staining was conducted using combinations of the indicated mAbs. Briefly, adherent cells in the culture plate were gently washed with PBS and were detached with 0.05% Trypsin-EDTA solution (Sigma, St Louis, MO, USA) at 37°C for five minutes. It was difficult to detach intact osteoclasts but a part of the osteoclasts could be analyzed by flow cytometry on Day 5. Cells were first incubated with Fc Block for mice or Fc(gamma)R-Binding Inhibitor for human, and then with an optimal dilution of biotinylated mAbs. After washing with 2% FBS in PBS, the cells were incubated with FITC- or PE-labeled mAbs or streptavidin, and also stained with 7-amino-actinomycin D (BD Pharmingen, San Jose, CA, USA) to exclude dead cells. After washing, the cells were analyzed on FACScan or FACScaliber (BD Bioscience) and analyzed with CellQuest (BD Bioscience).

#### Reverse transcription-polymerase chain reaction (RT-PCR)

Total RNA was isolated using STAT60 (Tel-Test, Friendwood, TX, USA) and was reverse transcribed to complementary DNA with oligo-dT and SuperScript RT (Invitrogen, Carlsbad, CA, USA). PCR consisted of 35 cycles of 45 seconds at 94°C, 1 minute at 58°C and 1 minute at 72°C. The primers were as follows: mouse Dll1 sense, 5'-ACCTTCTTTCGCGTATGCCTCAAG-3' and mouse Dll1 anti-sense, 5'-AGAGTCTGTATGGAGGGCTTC-3'; mouse Dll4 sense, 5'-CGAGAGCAGGGAAGCCATGA-3' and mouse Dll4 anti-sense 5'-CCTGCCTTATACCTCTGTGG-3'; mouse Jagged1 sense, 5'-ATTCGATCTACATAGCCTGTGAG-3' and mouse Jagged1 anti-sense 5'-CTATACGATGTATTCCATCCGGT-3'; mouse Jagged2 sense, 5'-TGTCAGCCACGGAGCAGTCATT-3' and mouse Jagged2 anti-sense 5'-TCTCACGTTCTTTCCTGCGCTT-3'; mouse beta-actin sense, 5'-GTGGGCCGCTCTAGGCACCAA-3' and mouse beta-actin anti-sense 5'-CTCTTTGATGTCACGCACGCACGATTTC-3'; human Dll1 sense, 5'-AGACGGAGACCATGAACAACCT-3' and human Dll1 anti-sense, 5'-CGTGGAAGTCCGCCTTCTT-3'; human Dll4 sense, 5'-TCAGCAAGATCGCCATCCA-3' and human Dll4 anti-sense 5'AGGGTGCTGGTTTGCTCATC-3'; human Jagged1 sense, 5'-GAGGCCGCCTCTCTGAACTCT-3' and human Jagged1 anti-sense 5'-CGATCTTGTTAGTAAACGTGATGGA-3'; human Jagged2 sense, 5'-AATGGAGTATTCTCGGATAGTTGCTATT-3' and human Jagged2 anti-sense 5'-GCACAACCTCTGGTAACAAACG-3'; human beta-actin sense, 5'-ATCTGGCACCACACCTTCTACAATGAGCTGCG-3' and human beta-actin anti-sense 5'-CGTCATACTCCTGCTTGCTGATCCACATCTGC-3'; mouse Hes-1 sense, 5'-CCGGTCTACACCAGCAACAGT-3' and mouse Hes-1 anti-sense 5'-CACATGGAGTCCGAAGTGAGC-3'; human Hes-1 sense, 5'-TCAACACGACACCGGATAAA-3' and human Hes-1 anti-sense 5'-TCAGCTGGCTCAGACTTTCA-3'. PCR products were separated by electrophoresis on 2.0% agarose gel with 0.5 μg/ml ethidium bromide and detected by UV.

#### Immunoblotting

Cells were lysed in SDS sample buffer (62.5 mM Tris-HCl, pH 6.8, 2% SDS, 20% sucrose, 0.02% pyronin G) and boiled. Samples were subjected to SDS polyacrylamide gel electrophoresis and transferred to a PVDF membrane. These membranes were incubated with diluted primary Abs in 5% Tris buffered saline/Tween 20 (BSA TBS/T) (10 mM Tris-HCl. pH 7.6, 50 mM NaCl, 0.1% Tween 20) overnight at 4°C. After washing with TBS/T, membranes were incubated with a horseradish peroxidase-conjugated secondary Ab. The immunoreactive proteins were visualized using ECL Plus (GE Healthcare, Chalfont St Giles, Buckinghamshire, UK).

#### Animal model of arthritis

Experimental arthritis was induced by passive transfer of serum from arthritic K/BxN mice, which spontaneously develop arthritis resembling RA [21]. These mice were kindly provided by Drs. C. Benoist and D. Mathis (Harvard Medical School, Boston, MA, USA). Preliminary experiments showed that injection of 100 μl of pooled serum into the peritoneal cavity on Day 0 and Day 2 induced arthritis consistently in C57BL/6 mice. Mice with arthritis were treated by intraperitoneal injection of 0.25 mg of anti-mouse Dll1 mAb (HMD1-5) or control hamster IgG (eBioscience) twice a week for two weeks. Treatment was begun on Day 3. Arthritis in each limb of arthritic mice was assessed clinically by visual scoring from 1 to 4: 0, no swelling; 1, detectable swelling in one joint; 2, non-severe swelling in two or more joints; 3, severe swelling in two or more joints; and 4, severe swelling in two or more joints including digital swelling. The maximal score for an individual animal was 16. Arthritis scores were analyzed statistically by Student's *t *test.

In histological examination, hindpaws were obtained and fixed in 10% buffered formalin, decalcified in 10% EDTA, and embedded in paraffin. Sections (4 μm) were stained with hematoxylin and eosin for histologic examination. All procedures met institutional regulations for animal experiments.

### Immunohistochemistry

Paraffin-fixed tissue sections of hindpaws were deparaffinized, pretreated in Liberate Antibody Binding Solution (Polysciences, Inc., Warrington, PA, USA) for five minutes, and incubated with 4 μg/ml of anti-TRAP Ab (K-17; Santa Cruz Biotechnology, Inc., Santa Cruz, CA, USA). They were then incubated with biotinylated Ab against goat immunoglobulins (DAKO Cytomation, Glostrup, Denmark), and ABC reagent (VectaStain Elite ABC kit; Vector Laboratories, Burlingame, CA, USA) was used for detection. Color was developed with diaminobenzidine, whereas the sections were counterstained with hematoxylin. Diaminobenzidine-positive MNCs in the metatarsal joints were enumerated by observers in a blind manner to calculate the TRAP-positive osteoclasts per bone surface. The numbers of TRAP-positive osteoclasts were analyzed statistically by Student's *t *test.

### Ovariectomy-induced bone loss

Eight-week-old female C57BL/6 mice were either sham-operated or ovariectomized (OVX) under anesthesia. 0.25 mg of anti-mouse Dll1 mAb (HMD1-5), anti-mouse Jagged1 mAb (HMJ1-29) or control hamster IgG was administered intraperitoneally twice a week for four weeks. At the end of the experiments, right and left femora were removed and fixed in 70% ethanol. The femoral bone was examined radiographically using quantitative computed tomography (CT) (LCT-200; LaTheta, Aloka, Tokyo, Japan) according to the manufacturer's instruction. CT scanning was performed at 96 μm intervals and 10 slices of trabecular bone were analyzed. Histomorphometric parameters for osteoclastogenesis were analyzed by staining of femoral bone sections with anti-TRAP Ab as mentioned above. Parameters obtained from microCT and histomorphometrical analysis were statistically analyzed by Student's *t *test. All procedures met institutional guidelines for animal experiments.

## Results

### Expression of Notch receptors and ligands during osteoclastogenesis from mouse BM

**Figure 1:**
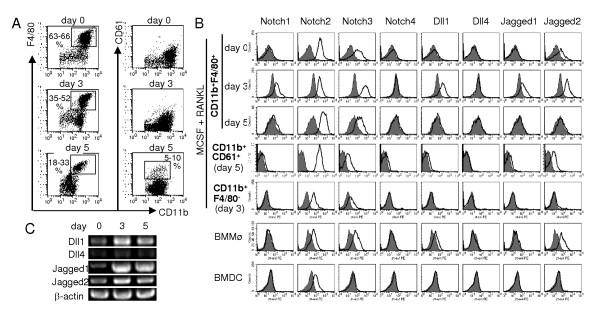
**Expression of Notch receptors and ligands during osteoclastogenesis from mouse BM**. BM cells cultured with rmM-CSF for 72 hours were used as osteoclast precursors (Day 0). These cells were further cultured with rmM-CSF and rhRANKL to induce osteoclasts. (**a**) CD11b^+^F4/80^+ ^osteoclast precursors and CD11b^+^CD61^+ ^osteoclasts were analyzed on days 0, 3 and 5. Percentages from three independent experiments are indicated. (**b**) Expression of Notch receptors and ligands on CD11b^+^F4/80^+ ^osteoclast precursors was analyzed by flow cytometry on days 0, 3 and 5. Expression on CD11b^+^F4/80^- ^cells on Day 3 is also represented. CD11b^+^CD61^+ ^osteoclasts were analyzed on Day 5. For comparison, BM cells were cultured with rmM-CSF alone for eight days and CD11b^+^F4/80^+ ^cells were analyzed as BM-derived macrophages (BMMø), or with rmGM-CSF for eight days with LPS stimulation for the last 17 hours and CD11c^+ ^cells were analyzed as BM-derived dendritic cells (BMDC). Filled histograms indicate the control hamster IgG. Open histograms indicate the Notch receptors or ligands. (**c**) Messenger RNA levels of Notch ligands were measured by RT-PCR on days 0, 3 and 5.

Mouse BM cells cultured with rmM-CSF for 72 hours were used as osteoclast precursors (Day 0). Flow cytometric analysis showed CD11b was expressed on more than 90% of cells during osteoclastogenesis induced with rmM-CSF and rhRANKL. Among these CD11b^+ ^osteoclast precursors, F4/80^+ ^cells differentiate into osteoclasts although the terminally differentiated osteoclasts do not express F4/80 [5,22]. As shown in Figure 1a, a substantial part of the CD11b^+ ^cells expressed F4/80 during osteoclastogenesis. Then, the expression of Notch receptors and ligands on CD11b^+^F4/80^+ ^cells were examined. CD11b^+^F4/80^+ ^osteoclast precursors expressed Notch2, Notch3 and Jagged2 on Day 0 (Figure 1b). Stimulation with rmM-CSF and rhRANKL induced Notch1, Dll1 and Jagged1 expression on Day 3 (Figure 1b). A part of these cells differentiated into CD11b^+^CD61^+ ^osteoclasts on Day 5 (Figure 1a). On Day 5, expression of Notch1, Dll1 and Jagged1 declined on CD11b^+^F4/80^+ ^cells, and on CD11b^+^CD61^+ ^osteoclasts (Figure 1b). CD11b^+^F4/80^- ^cells do not differentiate into osteoclasts but could potentially stimulate Notch receptors on CD11b^+^F4/80^+ ^cells. However, Notch ligands were not expressed on CD11b^+^F4/80^- ^cells while Notch2 and Notch3 were expressed on Day 3 (Figure 1b), as well as on Day 0 and Day 5 (data not shown). RT-PCR analysis during osteoclastogenesis also showed an increase of Dll1 and Jagged1 messenger RNA levels in total cells on Day 3, although they were still detected on Day 5 (Figure 1c). BM derived-macrophages, which were derived from BM cells by culturing with rmM-CSF alone, showed Notch receptor and ligand expression similar to osteoclast precursors at Day 0 (Figure 1b). In contrast, BM derived-dendritic cells expressed only Notch2 (Figure 1b).

### Dll1 enhances but Jagged1 suppresses osteoclastogenesis from mouse BM

**Figure 2:**
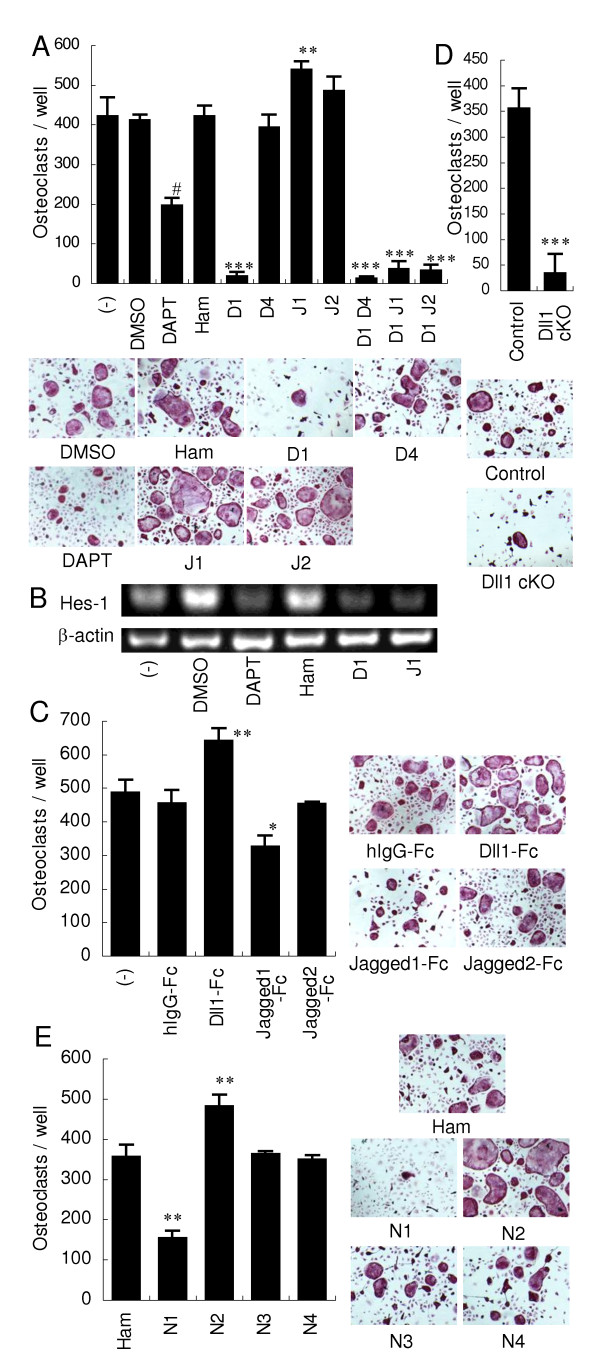
**Effects of Notch ligand blockade and Notch receptor stimulation on osteoclastogenesis from mouse BM**. (**a**) Osteoclasts were induced from mouse BM as described in Figure 1. DAPT (10 μM), DMSO as a control, 20 μg/ml of the indicated mAb (D1, HMD1-5; D4, HMD4-2; J1, HMJ1-29; J2, HMJ2-1), or control hamster IgG (Ham) was added on Day 0 and Day 3. (**b**) Osteoclasts were induced as described in (a) and mRNA levels of Hes-1 and beta-actin were determined by RT-PCR on Day 3. (**c **and **e**) Osteoclast precursors were pre-incubated with Fc Block and then differentiated on the plate immobilized with 10 μg/ml of the indicated Fc chimera or control human IgG-Fc fragment (hIgG-Fc) (c), or with 5 μg/ml of the indicated mAb (N1, HMN1-12; N2, HMN2-29; N3, HMN3-133; N4, HMN4-14) or Ham (e). (**d**) BM cells from Dll1-conditional knockout or littermate control mice were differentiated into osteoclasts. ***, *P *< 0.001. (a, c-e) Osteoclasts were stained for TRAP. Representative stainings are shown. Original magnification: × 100. Data are indicated as the mean TRAP-positive MNCs per culture well ± SD of triplicated wells. #, *P *< 0.001 versus DMSO control. *, *P *< 0.05; **, *P *< 0.01; ***, *P *< 0.001 versus Ham or hIgG-Fc control. Similar results were obtained in three independent experiments.

Since both Notch receptors and ligands were expressed on CD11b^+^F4/80^+ ^osteoclast precursors during osteoclastogenesis, we examined the role of Notch ligands. As shown in Figure 2a, blocking of Notch signaling by a gamma-secretase inhibitor, DAPT, significantly inhibited RANKL-induced osteoclastogenesis. Anti-mouse Dll1 blocking mAb also markedly inhibited the osteoclastogenesis. Anti-mouse Dll4 blocking mAb had no effect on osteoclast differentiation, consistent with no expression of Dll4 during osteoclastogenesis. Anti-mouse Jagged1 blocking mAb significantly enhanced osteoclastogenesis. Anti-mouse Jagged2 blocking mAb slightly enhanced osteoclastogenesis but not significantly. Blocking of Notch signaling with anti-mouse Dll1 or anti-mouse Jagged1 mAb, as well as DAPT, was shown by the inhibition of Hes-1 expression (Figure 2b). Combinational blockade of Dll1 with either Dll4, Jagged1 or Jagged2 also markedly decreased the osteoclastogenesis (Figure 2a). These results indicate that Dll1 plays a predominant role among Notch ligands in supporting osteoclast differentiation from mouse BM.

To confirm the effect of each Notch ligand on osteoclastogenesis, Notch receptors on osteoclast precursors were activated by Fc fusion protein of each Notch ligand. Accordingly, mouse Dll1-Fc significantly enhanced the osteoclastogenesis, whereas mouse Jagged1-Fc suppressed it (Figure 2c). Moreover, Dll1-deficient BM cells showed a greatly reduced osteoclastogenesis as compared with control cells (Figure 2d). Collectively, these results indicate that Dll1 enhances but Jagged1 suppresses osteoclastogenesis.

### Notch2 promotes but Notch1 suppresses osteoclastogenesis from mouse BM

To investigate if the activation of each Notch receptor enhances or suppresses osteoclast differentiation, osteoclast precursors from mouse BM were differentiated on the culture plate immobilized with mAb against each Notch receptor. Osteoclastogenesis was enhanced by anti-Notch2 agonistic mAb while it was suppressed by anti-Notch1 agonistic mAb (Figure 2e). The activation of Notch3 did not show any effect, though it was expressed during osteoclastogenesis (Figures 1b and 2e). Notch4 was not expressed during osteoclastogenesis and anti-mouse Notch4 mAb had no effect on osteoclast differentiation (Figures 1b and 2e). These results indicate that Notch2 enhances, but Notch1 suppresses, osteoclastogenesis.

### Expression of Notch receptors and ligands during osteoclastogenesis from human PBmono

**Figure 3:**
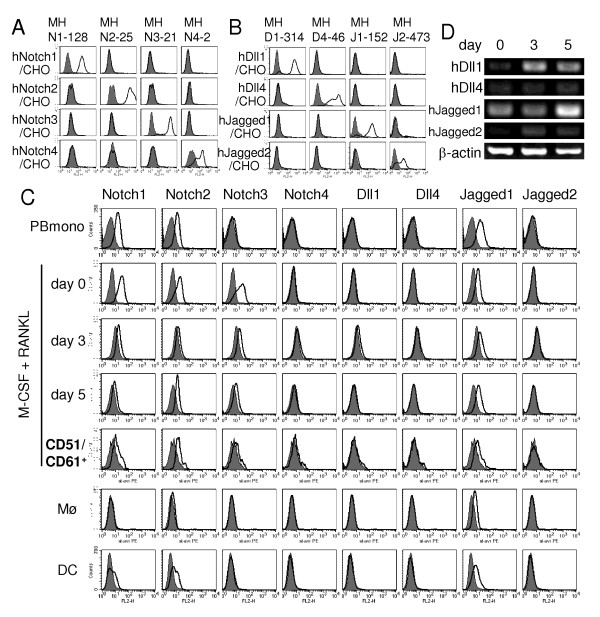
**Expression of Notch receptors and ligands during osteoclastogenesis from human PBmono**. Reactivity of anti-human Notch receptor mAbs (**a**) or anti-human Notch ligand mAbs (**b**) against CHO transfectants. Open histograms indicate the staining with anti-Notch1 (MHN1-128), anti-Notch2 (MHN2-25), anti-Notch3 (MHN3-21), anti-Notch4 (MHN4-2), anti-Dll1 (MHD1-314), anti-Dll4 (MHD4-46), anti-Jagged1 (MHJ1-152) or anti-Jagged2 (MHJ2-473) mAb. Filled histograms indicate the staining with control mouse IgG (a-c). (**c**) PBmono cultured with rhM-CSF for 72 hours were used as osteoclast precursors (Day 0). These cells were further cultured with rhM-CSF and rhRANKL to induce osteoclasts. CD14^+ ^cells were analyzed by flow cytometry on days 0, 3 and 5. CD51/CD61^+^CD14^+ ^osteoclasts were analyzed on Day 5. For comparison, PBmono were cultured with rhM-CSF alone for eight days and CD14^+ ^cells were analyzed as macrophages (Mø), or with rhGM-CSF and rhIL-4 for seven days with LPS stimulation for the last 48 hours and CD11c^+ ^cells were analyzed as dendritic cells (DC). Open histograms indicate the staining with MHN1-128 (Notch1), MHN2-25 (Notch2), MHN3-21 (Notch3), MHN4-2 (Notch4), MHD1-314 (Dll1), MHD4-46 (Dll4), MHJ1-152 (Jagged1), or MHJ2-473 (Jagged2). (**d**) Osteoclasts were induced as described in (c). Messenger RNA levels of Notch ligands were measured by RT-PCR on days 0, 3 and 5.

To study the role of Notch receptors and ligands in human osteoclastogenesis, we established mAbs specific for human Notch1, Notch2, Notch3, Notch4, Dll1, Dll4, Jagged1 and Jagged2 (Figure 3a, b). Then, the expression of Notch receptors and ligands during osteoclast differentiation from human PBmono were determined by flow cytometry. More than 92% of cells expressed CD14 during osteoclastogenesis induced with rhM-CSF and rhRANKL, which were analyzed as osteoclast precursors. 5 to 10% of CD14^+ ^cells expressed CD51/CD61 on Day 5, which were analyzed as osteoclasts. While Notch1 and Notch2 were expressed on PBmono, Notch3 expression was induced by rhM-CSF stimulation for 72 hours (Figure 3c, Day 0). The expression of Notch1, Notch2 and Notch3 was maintained during osteoclastogenesis, including CD14^+^CD51/CD61^+ ^osteoclasts on Day 5 although the expression levels were higher on Day 0 (Figure 3c). Similarly to the mouse BM, induction of Dll1 expression on CD14^+ ^osteoclast precursors was observed on Day 3 of RANKL stimulation, albeit at a low level. Meanwhile, Jagged1 was constitutively expressed (Figure 3c). RT-PCR analysis during osteoclastogenesis also showed the increase of Dll1 at messenger RNA level on Day 3 in total cells (Figure 3d). Monocyte-derived macrophages, which were induced with rhM-CSF alone, expressed only Notch2 and Jagged1 (Figure 3c). Monocyte-derived dendritic cells expressed Notch1, Notch2 and Jagged1 as did PBmono (Figure 3c).

### Dll1 enhances but Jagged1 suppresses osteoclastogenesis from human PBmono

**Figure 4:**
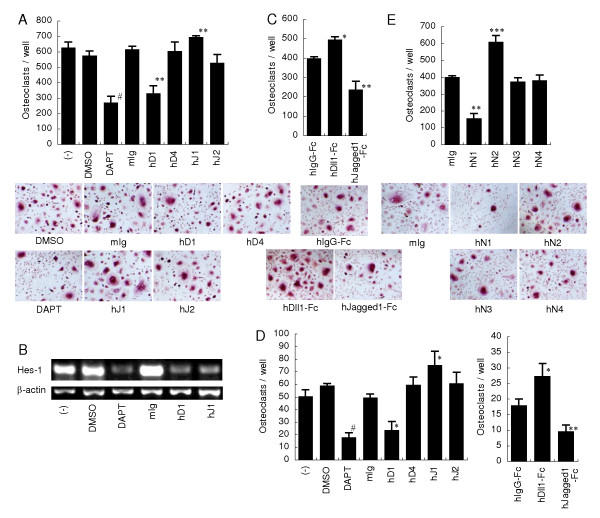
**Effects of Notch ligand blockade and Notch receptor stimulation on osteoclastogenesis from human PBmono**. (**a**) Osteoclasts were differentiated from human PBmono as described in Figure 3. DAPT (10 μM), DMSO as a control, 20 μg/ml of the indicated mAb (hD1, MHD1-314; hD4, MHD4-46; hJ1, MHJ1-152; hJ2, MHJ2-473), or control mouse IgG (mIg) was added on Day 0 and Day 3. (**b**) Osteoclasts were induced as described in (a) and mRNA levels of Hes1 and beta-actin were determined by RT-PCR. (**c **and **e**) Osteoclast precursors were pre-incubated with Fc(gamma)R-binding inhibitor and then differentiated on the culture plate immobilized with 10 μg/ml of the indicated Fc chimera or control human IgG-Fc fragment (hIgG-Fc) (c), or with 5 μg/ml of the indicated mAb (hN1, MHN1-128; hN2, MHN2-25; hN3, MHN3-21; hN4, MHN4-2) or mIg (e). (**d**) Osteoclasts were differentiated as described in (a) or (c) in the presence of a low dose of rhRANKL (12.5 ng/ml). (a, c-e) Osteoclasts were stained for TRAP. Representative stainings are shown (a, c and e). Original magnification: ×100. Data are indicated as the mean TRAP-positive MNCs per culture well ± SD of triplicated wells..#, *P *< 0.001; versus DMSO control. *, *P *< 0.05; **, *P *< 0.01; ***, *P *< 0.001 versus mIg or hIgG-Fc control. Similar results were obtained in three independent experiments.

To determine the role of Notch ligands in osteoclast differentiation from human PBmono, Notch ligands were blocked by mAbs specific for human Dll1, Dll4, Jagged1 or Jagged2. Similar to the effect on osteoclastogenesis from mouse BM, DAPT significantly inhibited the differentiation of osteoclasts from human PBmono (Figure 4a). Blockade of Dll1 also significantly inhibited the differentiation of osteoclasts (Figure 4a). In contrast, blockade of Jagged1 significantly enhanced the osteoclast differentiation, while blockade of Dll4 or Jagged2 had no significant effect (Figure 4a). Suppression of Hes-1 expression with anti-human Dll1 or anti-human Jagged1 mAb, as well as DAPT, showed the inhibition of Notch signaling (Figure 4b). Conversely, stimulation with immobilized human Dll1-Fc enhanced the osteoclastogenesis, while human Jagged1-Fc suppressed it (Figure 4c). Decreasing the dose of RANKL clearly showed the enhancement of osteoclastogenesis by blockade of Jagged1, as well as by stimulation with human Dll1-Fc (Figure 4d). These results indicate that Dll1 positively regulates the osteoclastogenesis from PBmono but Jagged1 regulates it negatively.

### Notch2 promotes but Notch1 suppresses osteoclastogenesis from human PBmono

Then, we determined the effect of each Notch receptor activation on the differentiation of osteoclasts from human PBmono. Similarly to the mouse BM (Figure 2e), osteoclastogenesis was significantly enhanced by stimulation with anti-Notch2 mAb but suppressed by anti-Notch1 mAb, while anti-Notch3 or anti-Notch4 mAb had no effect (Figure 4e). These results suggest that Notch2/Dll1 interaction promotes the differentiation of osteoclasts while Notch1/Jagged1 interaction suppresses it in both mice and humans.

### Preferential activation of Notch2 or Notch1 signaling by Dll1 or Jagged1 stimulation

**Figure 5:**
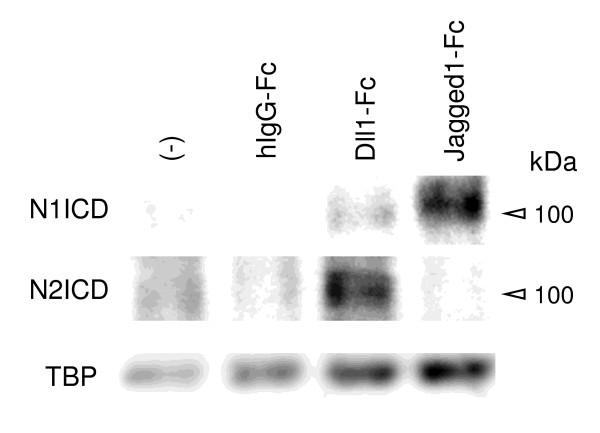
**Preferential activation of Notch2 or Notch1 by the stimulation with Dll1-Fc or Jagged1-Fc**. Osteoclasts were differentiated from mouse BM as described in Figure 2c. ICDs of Notch1 and Notch2 on day 1 were analyzed by Western immunoblotting. TBP was used as a loading control. N1, Notch1; N2, Notch2. Similar results were obtained in three independent experiments.

To address whether Notch1 preferentially interacts with Jagged1 and Notch2 preferentially interacts with Dll1, activation of Notch1 and Notch2 by Dll1-Fc or Jagged1-Fc stimulation during osteoclastogenesis was examined by immunoblotting of Notch1 and Notch2 intracellular domains (ICDs). As shown in Figure 5, Notch1 ICD was preferentially induced by the stimulation with Jagged1-Fc. On the other hand, Notch2 ICD was preferentially induced by the stimulation with Dll1-Fc. These results indicate the preferential interactions of Notch1/Jagged1 and Notch2/Dll1 during osteoclastogenesis.

### Blockade of Dll1 ameliorates arthritis and reduces osteoclasts in the affected joints

**Figure 6:**
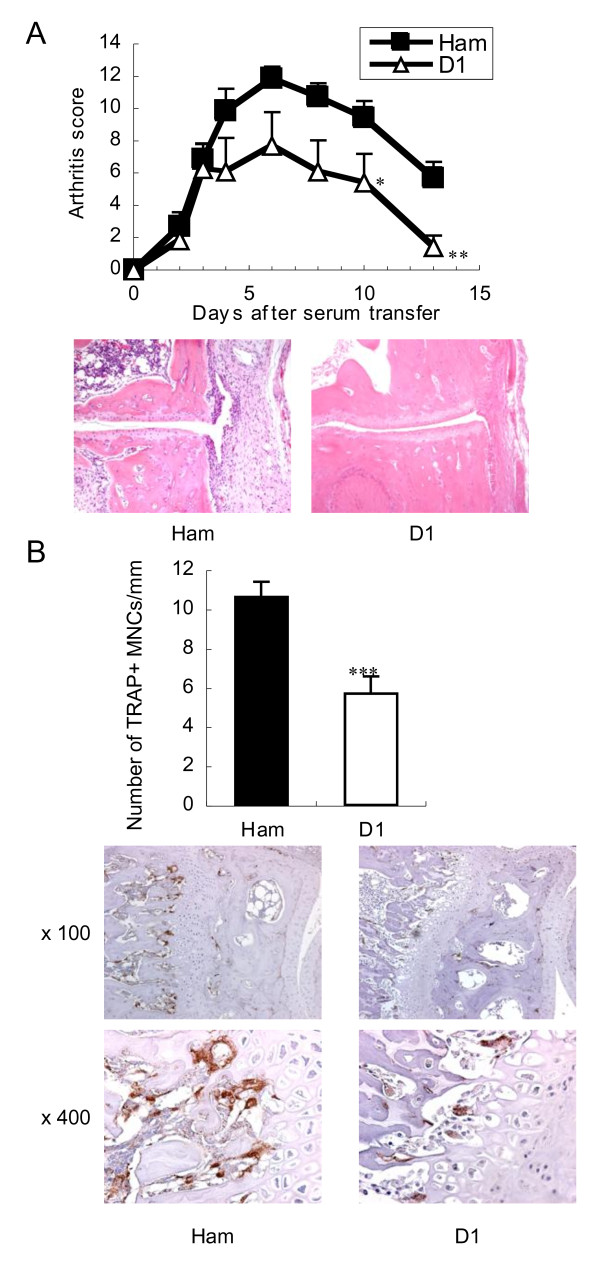
**Amelioration of arthritis and reduction of osteoclast numbers in the affected joints by blockade of Dll1**. Mice with K/BxN serum-induced arthritis were treated with control hamster IgG (Ham) or HMD1-5 (D1). (**a**) Severity of arthritis was assessed by an arthritis score. Mean score ± SEM is shown (*n *= 7 mice per group). *, *P *< 0.05; **, *P *< 0.01 versus control hamster IgG. Representative hematoxylin and eosin staining of joints on Day 13 is shown. Original magnification ×100. Similar results were obtained in two independent experiments. (**b**) TRAP immunohistochemistry of joints. Number of TRAP+ MNCs in joints on Day 13 was measured. Shown are the mean number of TRAP+ MNCs relative to bone surface ± SEM of 10 joints from 5 mice per group. ***, *P *< 0.001 versus control hamster IgG. Representative staining is shown. Original magnification; ×100, ×400.

Osteoclasts are known to play a pivotal role in the pathogenesis of bone erosion in RA, and our data indicated that osteoclastogenesis was dominantly supported by Dll1 among Notch ligands. In order to explore the role of Dll1 at the effecter phase of arthritis, K/BxN serum-induced arthritic mice, a mouse model for RA, were therapeutically treated with anti-mouse Dll1 mAb. K/BxN T cell receptor-transgenic mice produce high-titer arthritogenic autoantibody and spontaneously develop erosive polyarthritis resembling RA [21]. Passive transfer of serum containing arthritogenic autoantibody from K/BxN mice into normal mice also induces arthritis [23,24]. This serum-induced arthritis model makes it possible to focus on addressing the effecter phase of arthritis so that it is induced without the induction phase, including complicated immune responses. Actually, arthritis score and hematoxylin and eosin staining demonstrated that anti-mouse Dll1 mAb treatment ameliorated K/BxN serum-induced arthritis as compared to control hamster IgG treatment (Figure 6a). These arthritic mice developed joint deformity resembling RA, which can be determined visually. The incidence of joint deformity was decreased by the Dll1 blockade to 7.14% as compared to 57.1% of control (data not shown). TRAP immunohistochemistry showed that TRAP^+ ^multinucleated osteoclasts in the affected joints were significantly reduced by the Dll1 blockade (Figure 6b).

### Blockade of Dll1 suppresses OVX-induced bone loss

**Figure 7:**
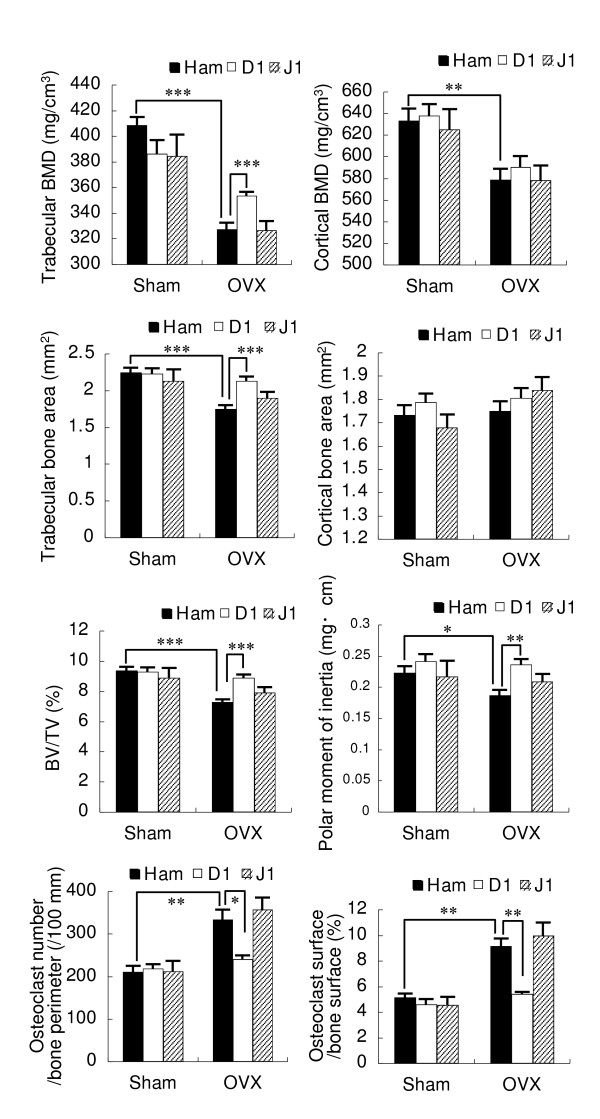
**Effect of Dll1 blockade on OVX-induced bone loss in mice**. OVX or sham-operated mice were treated with control hamster IgG (Ham), HMD1-5 (D1) or HMJ1-29 (J1). Trabecular and cortical BMD, trabecular and cortical bone area, bone volume per tissue volume (BV/TV), polar moment of inertia in the distal femur, osteoclast number/bone perimeter, and osteoclast surface/bone surface were evaluated four weeks after sham operation or OVX. Shown are the mean value ± SEM of 10 femora from 5 mice per group. *, *P *< 0.05; **, *P *< 0.01; ***, *P *< 0.001.

The reduction of osteoclasts in the affected joints of arthritic mice by the Dll1 blockade might be a result from the reduced inflammation. To investigate the direct effect of Dll1 blockade on osteoclastogenesis *in vivo*, the effect of anti-mouse Dll1 mAb on OVX-induced bone loss, a mouse model of osteoporosis, was evaluated. Trabecular and cortical bone mineral density (BMD) were markedly reduced in OVX mice. The anti-mouse Dll1 mAb treatment significantly increased trabecular BMD of OVX mice as compared to the control hamster IgG treatment although it had no effect on sham-operated mice (Figure 7). Meanwhile, the Dll1 blockade was not affected on cortical BMD of OVX and sham-operated mice as compared to the control hamster IgG treatment (Figure 7). OVX-induced reduction of trabecular bone area, as well as trabecular bone volume/tissue volume (BV/TV), was also significantly prevented by the Dll1 blockade while OVX had no effect on cortical bone area (Figure 7). In addition, the Dll1 blockade protected against OVX-induced reduction of polar moment of inertia, a parameter of bone strength that described the resistance to twisting (Figure 7). Histomorphometric parameters (osteoclast number/bone perimeter and osteoclast surface/bone surface) revealed suppression of osteoclastogenesis by the Dll1 blockade in OVX mice (Figure 7). These data indicate that blockade of Dll1 improves OVX-induced bone loss. Notably, the Jagged1 blockade did not show significant effect on these parameters of OVX and sham-operated mice (Figure 7).

## Discussion

In this study, we demonstrated that mouse osteoclast precursors expressed multiple Notch receptors and ligands during osteoclastogenesis, but Notch2/Dll1 axis enhanced and Notch1/Jagged1 axis suppressed osteoclastogenesis selectively. A similar regulation of osteoclastogenesis by Notch2/Dll1 and Notch1/Jagged1 axes was also demonstrated in humans. Finally, we showed that blockade of Dll1 could suppress osteoclastogenesis in the affected joints in a murine arthritis model.

The inhibition of osteoclastogenesis by Dll1 blockade and the enhancement by stimulation with Dll1-Fc and anti-Notch2 mAb suggest that Dll1 promotes osteoclastogenesis via Notch2. On the other hand, the enhancement of osteoclastogenesis by Jagged1 blockade and the inhibition by stimulation with Jagged1-Fc and anti-Notch1 mAb suggest that Jagged1 suppresses osteoclastogenesis via Notch1. The preferential induction of Notch1 ICD by Jagged1-Fc stimulation and that of Notch2 ICD by Dll1-Fc stimulation supported this notion. Although we could not directly indicate the preferential Dll1/Notch2 and Jagged1/Notch1 interactions due to a lack of appropriate blocking mAbs against Notch1 and Notch2, such a preferential Notch2/Dll1 interaction also plays a key role in the development of marginal zone B cells in the spleen [25] and a preferential Notch1/Jagged1 interaction has been implicated in the maintenance of hematopoietic stem cells in the BM [26,27]. It has been known that interaction of Notch receptors with Dll versus Jagged ligands is affected by glycosylation of Notch extracellular domain by Fringe [28,29]. Therefore, a differential modification of Notch1 and Notch2 on osteoclast precursors by Fringe or a differential modification of Notch1 and Notch2 interactions with Jagged1 and Dll1 by Fringe might be responsible for the preferential Notch2/Dll1 and Notch1/Jagged1 interactions. Further studies are needed to address these possibilities.

The enhancement of osteoclastogenesis by stimulation with anti-Notch2 mAb and the suppression by anti-Notch1 mAb suggest a differential signaling via Notch1 versus Notch2. The inhibition of osteoclastogenesis by blockade of net Notch signaling by DAPT implies that the promotion via Notch2 is dominant over the suppression via Notch1 during osteoclastogenesis. The pro-osteoclastogenic function of Notch2 is consistent with a previous report demonstrating that silencing Notch2 with small hairpin RNA suppressed osteoclastogenesis and overexpression of Notch2 intracellular domain enhanced it [4]. The anti-osteoclastogenic function of Notch1 is also consistent with a previous report demonstrating that deletion of Notch1 in murine myeloid cells enhanced osteoclastogenesis and bone resorption [3]. Notch2 has been shown to act in conjunction with nuclear factor-kappaB, possibly by regulating the nuclear factor of activated T cells (NFAT)-c1 promoter during the terminal differentiation of osteoclasts [4]. In contrast, Jagged1-mediated Notch1 signaling could not cooperate with nuclear factor-kappaB but was likely to inhibit proliferation of osteoclast precursors [3].

Blockade of Dll1 suppressed the osteoclastogenesis not only *in vitro *but also in a murine arthritis model. Prevention of OVX-induced trabecular bone loss by the Dll1 blockade supported the effect *in vivo*. Notably, blockade of Dll1, as well as Jagged1, did not affect the parameters of bone strength and structure in the absence of any stimulation as shown in sham-operated mice. A high expression of Dll1 and Jagged1 as well as Notch1, Notch2 and Notch3 has been demonstrated in the synovium of RA patients [30,31]. We previously demonstrated that Dll1 was expressed on a part of the macrophage population and that inflammatory cytokines, such as tumor nuclear factor-alpha or interferon-gamma, induced the expression of Dll1 on macrophages [9]. Thus, Dll1 blockade may be a novel strategy to prevent bone erosion in RA patients by suppressing the inflammation-associated osteoclastogenesis. In addition, Dll1 has also been implicated in the development of pathogenic Th1 effector cells [32,33], while Jagged1 has been implicated in the development of Th2 or regulatory T cells [32,34,35]. Accordingly, we previously demonstrated that Dll1 blockade ameliorated experimental autoimmune encephalomyelitis while Jagged1 blockade exacerbated it [14]. Moreover, we recently demonstrated that Jgged1 blockade exacerbated collagen-induced arthritis [36]. Therefore, the blockade of Dll1/Notch2 axis and the enhancement of Jagged1/Notch1 axis may be beneficial for the treatment of RA through multiple mechanisms.

## Conclusions

We demonstrate that Dll1 promotes osteoclastogenesis via Notch2, while Jagged1 suppresses osteoclastogenesis via Notch1 in both mice and humans. Osteoclastogenesis is suppressed by inhibition of Notch signaling with a gamma-secretase inhibitor, implying that Notch2/Dll1-mediated enhancement is dominant. Notably, blockade of Dll1 with anti-Dll1 mAb in RA model mice ameliorates arthritis and reduces the number of osteoclasts in the affected joints. Prevention of OVX-induced trabecular bone loss by the Dll1 blockade supported the effect in osteoclastogenesis. We, therefore, propose that the differential regulation of osteoclastogenesis by Notch2/Dll1 and Notch1/Jagged1 axes could be a novel target for the treatment of RA to prevent bone erosion.

## Abbreviations

BM: bone marrow; BMD: bone mineral density; BV/TV: bone volume/tissue volume; CT: computed tomography; Dll, Delta-like; FITC: Fluorescein isothiocyanate; HAT: hypoxanthine aminopterin thymidine; ICD: intracellular domain; M-CSF: macrophage-colony stimulating factor; MNCs: multinucleated cells; mAb: monoclonal antibody; NFAT: nuclear factor of activated T cells; OVX: ovariectomized; PBmono, peripheral blood monocytes; PBS: phosphate-buffered saline; RA: rheumatoid arthritis; RANKL: receptor activator of nuclear factor-kappaB ligand; rh: recombinant human; rm: recombinant mouse; RT-PCR: reverse transcription-polymerase chain reaction TRAP: tartrate-resistant acid phosphatase.

## Competing interests

The authors declare that they have no competing interests.

## Authors' contributions

CS carried out the experiments and the analysis of data, participated in the design of the study and drafted the manuscript. AK participated in the generation of monoclonal antibodies. NK participated in the generation of monoclonal antibodies. KH participated in the generation of knockout mice. SC participated in the generation of monoclonal antibodies. HY conceived of the study, participated in its design and coordination, and edited the manuscript. All authors read and approved the final manuscript.
